# Contrast-Enhanced Ultrasound Imaging Quantification of Adventitial Vasa Vasorum in a Rabbit Model of Varying Degrees of Atherosclerosis

**DOI:** 10.1038/s41598-017-06127-w

**Published:** 2017-08-01

**Authors:** Xiaoying Li, Ruyou Zhang, Zongmin Li, Chunping Ning, Zhenzhen Wang, Meizheng Dang, Yanqing Peng, Xuesong Han, Litao Sun, Jiawei Tian

**Affiliations:** 1 The 2nd Affiliated Hospital of Harbin Medical University, Harbin, China; 2 Harbin the first hospital, Harbin, China; 3 The Affiliated Hospital of Qingdao University Medical College, Qingdao, China

## Abstract

This study used an atherosclerotic rabbit model to investigate the feasibility of quantifying adventitial vasa vasorum (VV) via contrast-enhanced ultrasound (CEUS) imaging to identify early atherosclerosis. Recent evidence has linked adventitial VV with atherosclerotic plaque progression and vulnerability. A growth in VV density has been detected preceding intimal thickening and even endothelial dysfunction. In our study, carotid atherosclerosis rabbit models were used, and animals underwent CEUS imaging at the end of the atherosclerotic induction period. Normalized maximal video-intensity enhancement (MVE) was calculated to quantify VV density. After CEUS imaging, animals were euthanized, and their carotids were processed for histopathological analysis following staining for CD31 and VEGF. Adventitial normalized MVE increased as atherosclerosis progressed (*p* < 0.001), and normalized MVE also progressed, demonstrating a linear correlation with histological findings (r = 0.634, *p* < 0.001 for VEGF-positive; r = 0.538, *p* < 0.001 for CD31-positive). Thus, we histologically validated that CEUS imaging can be used to quantify the development of adventitial VV associated with atherosclerosis progression. This method can be used for monitoring the VV to detect early atherosclerosis.

## Introduction

Worldwide, stroke is the second most common cause of death after ischaemic heart disease^1, 2^. Carotid stenosis caused by the formation and rupture of atherosclerotic plaques is a major risk factor for ischaemic stroke^3^. The vasa vasorum (VV) is a network of small blood vessels located in the adventitia of large arteries. Neomicrovessels sprouting from the adventitial VV network result in intraplaque neovascularization^4^. The extent of adventitial neovascularization is directly associated with plaque vulnerability^5–7^. Previous studies have demonstrated that there is a significant correlation between the intensity of intraplaque haemorrhages and the density of VV neovascularization and bleeding^8, 9^. Recent data have indicated that increases in VV density were preceded by intimal thickening and endothelial dysfunction, suggesting that neoangiogenesis could occur at the early stages of atherogenesis^10–12^. Therefore, observations of neovascularization and adventitial VV are important to identify atherosclerotic plaque development and stability.

Early detection of intraplaque neovascularization using a non-invasive technique could help identify a high-risk plaque^13^. Imaging techniques such as coronary angiography, intravascular ultrasound and MRI have their advantages; however, they are often limited by complex operation, higher cost, and limited ability to characterize plaque composition^14, 15^. Recently, contrast-enhanced ultrasound (CEUS) has emerged as a feasible and effective method for imaging the VV^16–18^. Some studies detected visualization of contrast microbubbles within plaque neovascularization^19^. Moreover, these findings were confirmed by a more recent study that used CEUS imaging of adventitial VV in carotid arteries to check the presence and degree of intraplaque neovascularization; these findings were corroborated by the histology of the same lesions^17, 20^. Because carotid endarterectomy specimens do not include the entire arterial wall and because there is a possibility of high-risk lesions being missed during sample collection, histology cannot entirely confirm the imaging results despite the promising potential application of CEUS imaging in the clinical setting. In addition, further validation of CEUS imaging to quantitate VV density over the time course of plaque evolution is indispensable to its clinical application.

The current study was designed to investigate the feasibility of quantitative CEUS imaging for the *in vivo* visualization of intraplaque and adventitial neovascularization during atherosclerosis progression. Serial CEUS imaging was performed in an experimental carotid atherosclerotic rabbit model, with systematic histological assessment as the reference standard.

## Methods

### Animals

All rabbits (New Zealand white male rabbits) were obtained from the model animal centre of the 2^nd^ Affiliated Hospital of Harbin Medical University. The principles of laboratory animal care were followed, and all procedures were conducted according to the guidelines established by the National Institutes of Health, with efforts made to minimize suffering. The study protocol was approved by the Medical Ethics Committee on Animal Research of the 2^nd^ Affiliated Hospital of Harbin Medical University (Ethics No. KY2016-090).

### Carotid atherosclerotic animal model

**Figure 1:**
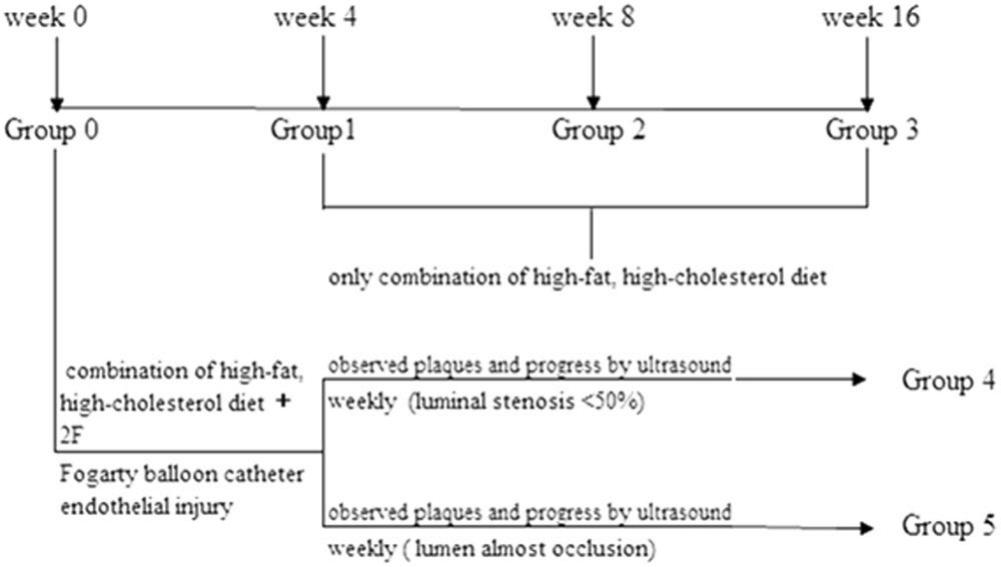
Rabbit model groups.

Carotid atherosclerosis was induced in New Zealand white adult male rabbits (2.5–3.5 kg) (n = 30) by feeding the rabbits a high-fat diet [1% cholesterol (Shanghai Lanji technology), 10% lard (Shandong Shiyuantianjiaji Factory), and 3% yolk powder (Shandong Shiyuantianjiaji Factory)] for 4 weeks, 8 weeks, or12 weeks, and these animals were assigned to groups 1, 2 and 3, respectively. An accelerated atherosclerotic rabbit model (n = 20) was generated by feeding the rabbits a combination of a high-fat diet (1% cholesterol, 10% lard, and 3% yolk powder) and an endothelial injury caused by a 2 F Fogarty balloon catheter (Boston Scientific, Temecula, California). The progression of plaques in these rabbits was observed by weekly 2D-ultrasound examinations, and the rabbits were divided into two additional groups (group 4: small plaque luminal stenosis <50% and group 5: large plaque lumen, almost occlusion). Age-matched rabbits (n = 10) maintained on normal chow served as the control group (group 0) (Fig. 1).

Atherosclerosis was initiated after one week of this combination of high-fat diet. Rabbits were anaesthetized with ketamine (35 mg/kg intramuscularly), xylazine (5 mg/kg intramuscularly) and acepromazine (0.75 mg/kg intramuscularly). Anaesthesia was maintained during the procedure through isoflurane inhalation. The right carotid arteries were injured with a balloon catheter, as described in a previous study^21^. Briefly, the balloon catheter was gently advanced into the right common carotid artery through the external carotid artery. The balloon was gently inflated at 2 atm and retracted. This procedure was repeated three times in each rabbit. The balloon catheter was then removed, the incision was closed with a suture, and the rabbits were allowed to recover.

### Contrast ultrasound imaging

CEUS examinations were performed using an advanced ultrasound equipment (HITACHIHI VISION Preirus, Hitachi Ltd., Tokyo, Japan) and ultrasound contrast software. Contrast pulse sequencing is a multi-pulse imaging method utilizing phase and amplitude modulation of transmission ultrasound combined with cancellation algorithms to detect microbubble-specific signals. A preliminary study was performed to establish the optimization of the contrast agent and image settings. CEUS imaging of the carotid artery was performed using USphere™ (with an average diameter of 1.2 µm, phospholipid-coated microbubbles, all fluorinated carbon gases, TRUST BIO SONICS) as the contrast agent, and vibration activation every 30 s by a special concussion instrument (For USphere™ series use only, TRUST BIO SONICS) before use. Rabbits were anaesthetized as described above, and a 50 µL contrast bolus was injected through an ear vein, followed by 1 mL normal saline injection. An EUP-L74M non-linear probe (5~13 HZ) with real-time ultrasound imaging (MI 0.15) was performed to capture microbubble reflow into the carotid artery lumen and the VV. To obtain satisfactory CEUS images, B-mode ultrasound scanning was set to reveal the details of the deeper wall adventitia interface, avoiding US noise in the vessel lumen, and to allow similar average greyscale levels of the deep and superficial regions^22^. The contrast-enhanced ultrasound images relied on 2D long-axis imaging of vessels and fully displayed the largest view of the carotid atherosclerotic plaque.

Digitally acquired ultrasound images were analysed off-line using customized CEUS imaging software by an observer blinded to the experimental conditions. Video-intensity in the region of interest (ROI) drawn in the plaque and adventitia of the injured vessel segment was measured over time using CEUS imaging software, and time-intensity curves were generated. Video-intensity data, which relate to the concentration of microbubbles in tissue, were plotted against the time elapsed from the destruction pulse. The maximal video-intensity enhancement (MVE) was the peak video-intensity from the time-intensity curves minus the background video-intensity. The normalized maximal video-intensity was calculated as the maximal video-intensity in the adventitia divided by the luminal region maximal video-intensity of interest drawn proximal to the lesion^23^. We traced different ROI for their MVE. The ROI may be located at areas of the carotid lumen, the atherosclerotic plaque, or the adventitia. The area of the ROI of the same carotid sample was used to obtain the MVE.

### Histology and immunohistochemistry

The carotid atherosclerotic rabbits (n = 22) at weeks 4, 8, and 12 were sacrificed by an overdose of intravenous sodium pentobarbital. The accelerated atherosclerotic rabbits with small atherosclerotic plaques that were characterized by luminal stenosis less than 50% and large atherosclerotic plaques that were characterized by full occlusion of the lumen were sacrificed by an overdose of intravenous sodium pentobarbital after confirmation by B model ultrasound imaging. The right carotid arteries were swiftly removed. Each specimen was fixed with 4% paraformaldehyde fixative and embedded in paraffin for haematoxylin and eosin (H&E) staining and immunostaining. Serial cross-sections with a thickness of 3 µm were stained with H&E and observed by light microscopy (Olympus, BX41, Tokyo, Japan). Specimens were immunostained with an anti-CD31 antibody (1:100 dilution; Abcam, Cambridge Science Park Cambridge, UK) and an anti-VEGF antibody (1:800 dilution; Abcam, Cambridge Science Park Cambridge, UK) for characterization and quantification of neovessels. Immunohistochemical reagents and secondary antibodies were from Maixin Bio and, specimens were visualized using a DAKO Envision System. The VV number was quantified by counting the total number of CD31-positive and VEGF-positive microvessels per carotid artery cross-section. The counting was performed by two independent observers, and the means of two values were used for analysis.

### Plasma lipid profile

Apolipoprotein A (APOA), apolipoprotein B (APOB), C-reactive protein (CRP), plasma total cholesterol (TC), triglyceride (TG), high-density lipoprotein (HDL), and low-density lipoprotein (LDL) levels were determined by enzymatic assays of blood samples, which were collected through an ear vein, and serum was separated by centrifugation for 15 min at 4 °C.

### Statistical analysis

All data analyses were performed using PASW 18.0 (IBM, New York, United States). P < 0.05 was considered statistically significant. The CEUS imaging parameters, serum lipid levels, and histological data are presented as the mean ± SD. The significance of the differences among all of the parameters in the two groups was tested using Student’s t-test. Differences in multiple groups were analysed using Friedman one-way ANOVA. The significance of the differences between the two groups was tested using Dunnett’s T3 test. The relationships between CEUS imaging and histological data were analysed by linear correlation analysis.

## Results

All rabbits successfully underwent CEUS imaging examinations except four (one on the combination diet from group 2, two from group 3 because of fatty diarrhoea, and one from group 5 because of severe carotid stenosis leading to stroke symptoms) who died halfway. Group 5 was designed for direct observations of adventitial and plaque VV; therefore, we could not calculate the normalized maximal video-intensity for this group.

### Plasma lipid profile

As expected, experimental groups 1–5 in our study had higher CRP levels than the control group. The ApoA levels in groups 2, 3, 4, and 5 were higher than the control. The ApoB levels in groups 3, 4, and 5 were higher than the control. The TC and LDL levels in groups 1–5 were all higher than the control. The TG levels in groups 3 and 5 were higher than the control. It is interesting to note that there were no significant differences among groups 1, 2, or 4 and the control for HDL levels. Furthermore the HDL levels in groups 3 and 4 were higher than the control. However, the HDL/TC ratio in groups 1–5 was all lower than the control. The results are shown in Table 1.

**Table 1:** The plasma lipid profile of every group.

Variables	CRP	ApoA	ApoB	TC	TG	LDL	HDL	HDL/TC
Group 0 (control)	1.218 ± 0.514	0.071 ± 0.050	0.102 ± 0.044	2.901 ± 2.788	1.457 ± 1.242	1.645 ± 2.068	0.522 ± 0.218	0.330 ± 0.251
Group 1	2.354 ± 0.477	0.078 ± 0.043	0.150 ± 0.060	18.948 ± 4.633	1.270 ± 0.796	8.276 ± 4.833	0.484 ± 0.154	0.0061 ± 0.082
p*	**0.001**	0.793	0.100	**<0.001**	0.766	**0.002**	0.735	**0.005**
Group 2	2.582 ± 1.352	0.126 ± 0.057	0.222 ± 0.193	22.226 ± 8.208	2.243 ± 1.673	10.722 ± 2.750	0.694 ± 0.176	0.033 ± 0.009
p**	**0.009**	**0.039**	0.072	**<0.001**	0.258	**0.001**	0.077	**0.003**
Group 3	4.352 ± 2.121	0.302 ± 0.104	1.556 ± 0.953	39.402 ± 1.914	9.500 ± 4.358	19.714 ± 4.459	1.463 ± 0.526	0.048 ± 0.018
p***	**0.001**	**<0.001**	**<0.001**	**<0.001**	**<0.001**	**<0.001**	**<0.001**	**<0.028**
Group 4	3.117 ± 2.121	0.129 ± 0.060	0.300 ± 0.328	24.813 ± 9.446	2.966 ± 3.805	11.414 ± 2.729	0.779 ± 0.345	0.035 ± 0.013
P****	**0.014**	**0.034**	**0.075**	**<0.001**	0.251	**<0.001**	0.067	**0.002**
Group 5	4.231 ± 2.567	0.240 ± 0.083	1.093 ± 1.153	34.769 ± 9.535	6.265 ± 4.410	15.977 ± 5.579	1.385 ± 0.708	0.038 ± 0.015
p*****	**0.002**	**<0.001**	**0.014**	**<0.001**	**0.004**	**<0.001**	**0.002**	**0.001**

p*: Group 1 VS Group 0; p**: Group 2 VS Group 0; p***: Group 3 VS Group 0; p****: Group 4 VS Group 0; p*****: Group 5 VS Group 0. *p* < 0.05 (indicated by bold face type) was considered statistically significant.

### CEUS imaging findings

**Figure 2:**
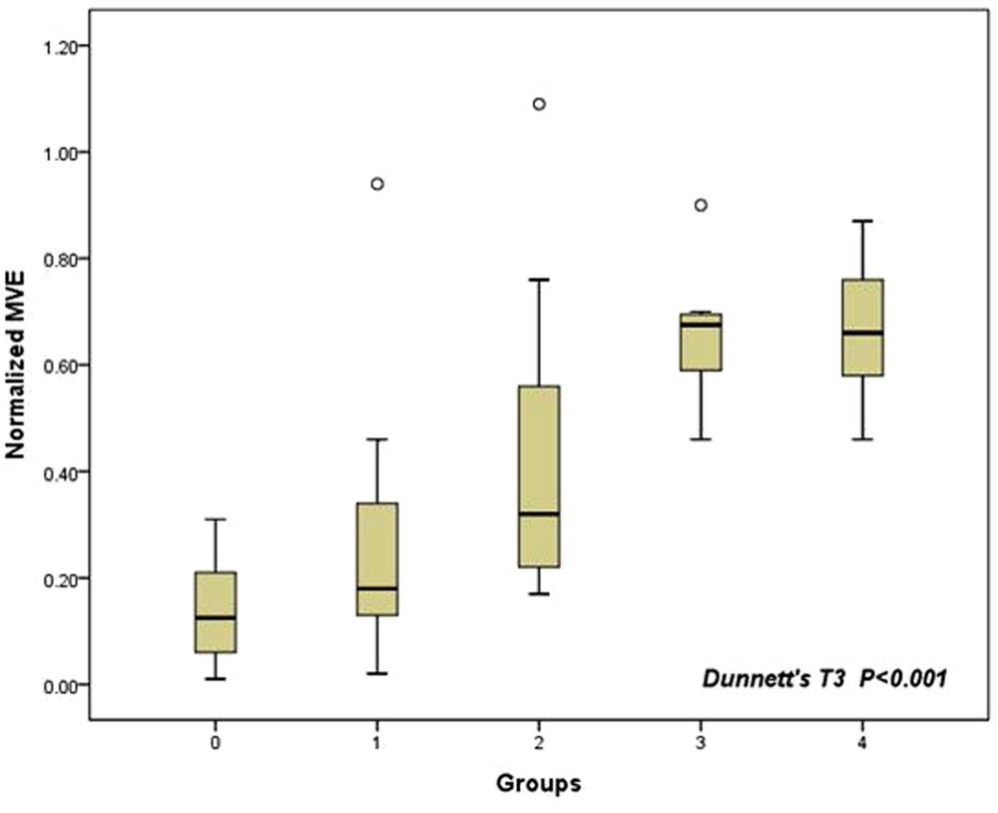
Normalized maximal video-intensity enhancement of the adventitial vasa vasorum in different groups.

The normalized MVE of the adventitial VV of groups 1–4 increased gradually compared to the control group (p < 0.001) (Fig. 2) (Group 0: 0.146 ± 0.099; group 1: 0.278 ± 0.267; group 2: 0.435 ± 0.313; group 3: 0.660 ± 0.127, group 4 0.660 ± 0.129). There was a significant difference among group 0 and groups 2, 3, and 4 (p = 0.025, p < 0.001, and p < 0.001 respectively).

In addition, we also compared a group of early atherosclerosis animals fed a high-fat diet without endothelial injury confirmed by pathology to the control group, and there was an increase in normalized MVE (0.146 ± 0.099 Vs 0.352 ± 0.293, p = 0.01).

**Figure 3:**
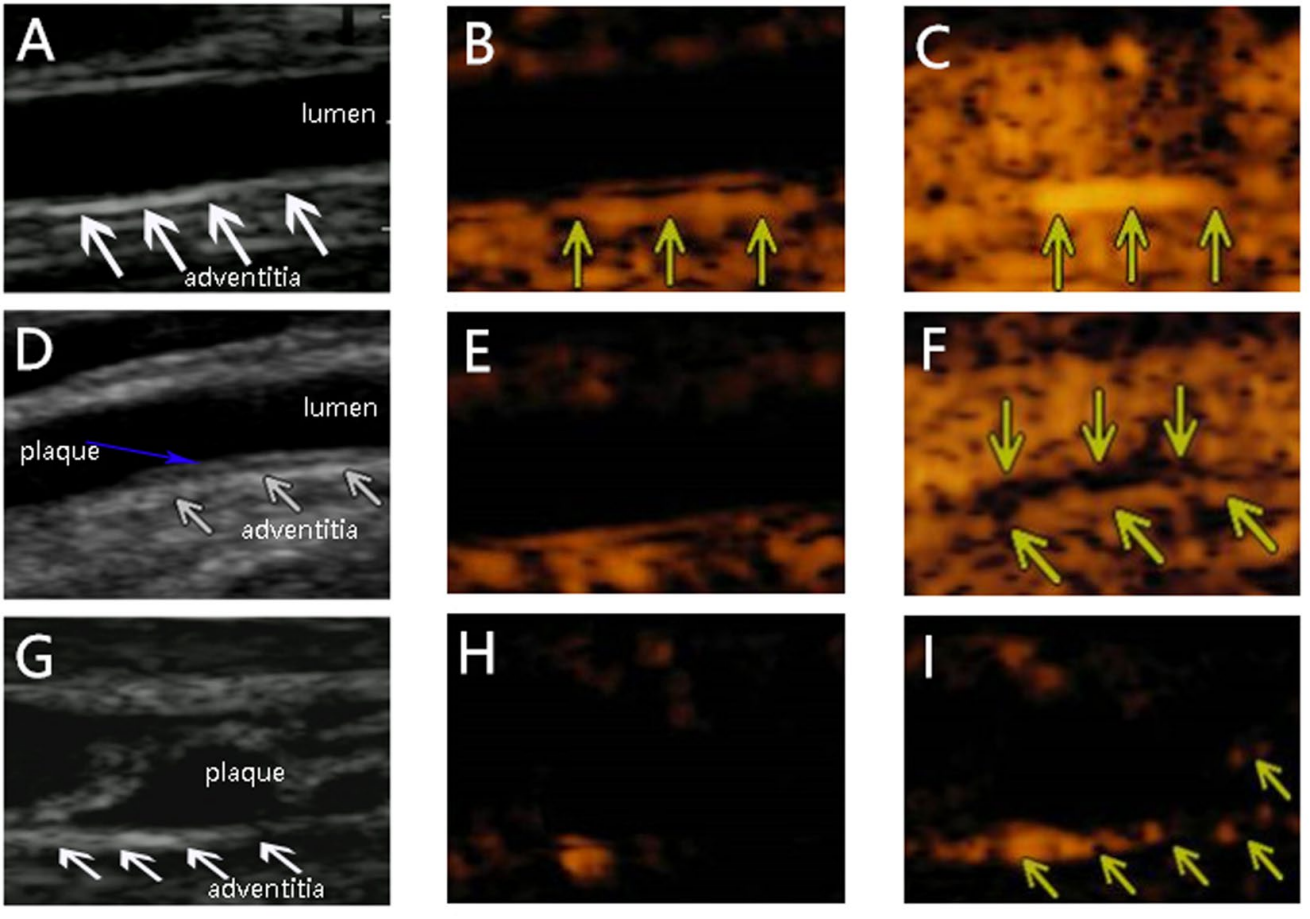
Two-dimensional ultrasound and contrast ultrasound images of the carotid before and after contrast injection. Two-dimensional ultrasound image of the carotid artery clearly shows the adventitia interface and lumen (**A**). The carotid artery lumen was dark before contrast injection, and the outer membrane is clearly visible as indicated by the yellow arrows (**B**). The carotid lumen became immediately visible, and the adventitial also showed enhanced signal after contrast injection, as indicated by the yellow arrows (**C**). There was a small plaque on the wall of the deep region as indicated by the blue arrows (**D**). The carotid artery lumen was dark before contrast injection (**E**). The plaque was a little filling defect, and the outer membrane was visible with more adventitial contrast as indicated by the arrows (**F**). There was a large mixed echo plaque almost filling the lumen (**G**). Before injection of contrast, the carotid artery lumen was dark (**H**). After contrast injection, the outer membrane was obviously enhanced and much more adventitial contrast was visible. There is contrast enhancement inside the plaque as indicated by the yellow arrows (**I**).

Figure 3 shows a representative consecutive carotid CEUS and two-dimensional ultrasound images from rabbits with varying degrees of atherosclerosis before and after CEUS imaging. The two-dimensional ultrasound image of the carotid artery demonstrated the interface between the adventitia and arterial lumen clearly (Fig. 3A). The arterial intima was thin and smooth. No plaque was detected. Before injection of contrast, the right carotid artery lumen was dark due to tissue signal suppression (Fig. 3B). After contrast injection, the carotid lumen was immediately enhanced, and the adventitial VV of the high-fat diet model animals also showed enhanced signals (Fig. 3C). Foam cells in the subintima and adventitial neovascularization proliferation were later confirmed by haematoxylin and eosin staining. Figure 3D–F were taken from a rabbit of group 4, which modelled accelerated atherosclerosis. We can see a small plaque on the intima of the posterior wall of the carotid artery (Fig. 3D) and a small filling defect (Fig. 3F) (Real-time CEUS imaging see video 1). More enhanced signals were detected in Fig. 3F than Fig. 3E. The three images were taken from an accelerated atherosclerotic rabbit in group 5. The lumen was almost occluded by a large plaque (Fig. 3G). The arterial lumen was dark before the injection of contrast agents (Fig. 3H). After the injection, the outer membrane was enhanced and much more adventitial contrast was visible than in the other groups (Fig. 3I). Online videos show the same findings in the form of higher resolution movie files.

### Histological findings

**Figure 4:**
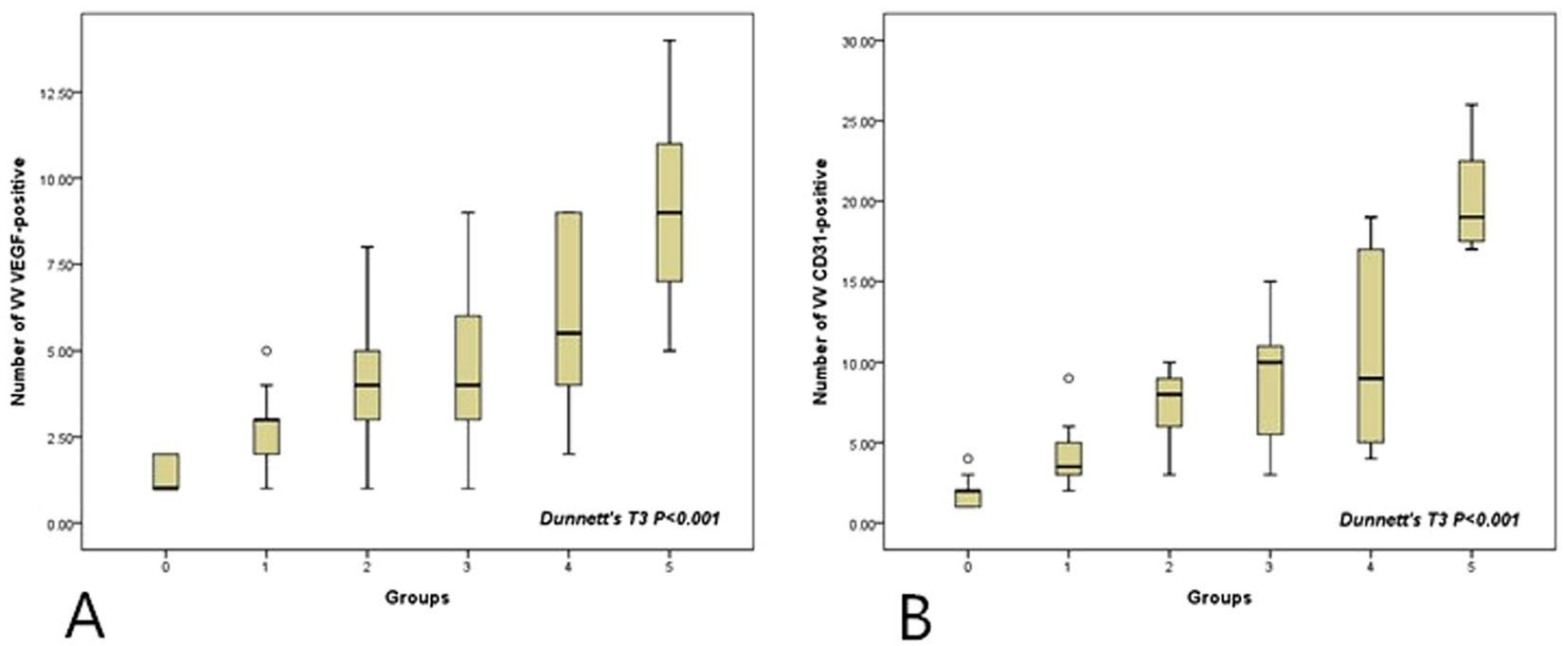
Number of VEGF-positive in the adventitial vasa vasorum from immunohistochemical analysis (**A**). Number of CD31-positive microvessels in the adventitial vasa vasorum from immunohistochemical analysis (**B**).

A gradual increase in VEGF-positive microvessels was observed in groups 1–5 compared to the controls, and the increase in group 5 with large plaques that almost occluded the lumen was very abrupt (p < 0.001) (Fig. 4A). Furthermore, differences in the number of VEGF-positive microvessels between the two accelerated atherosclerotic groups, groups 4 and 5, were significant (p = 0.023).

The number of CD31-positive microvessels showed a similar trend to VEGF-positive microvessels (Fig. 4B). A significant increase in CD31-positive microvessels was observed (p < 0.001). Compared to group 4 (accelerated atherosclerotic rabbit model with small plaques), a higher number of CD31-positive microvessels was observed in group 5 (p = 0.002).

The number of VEGF-positive adventitial microvessels in the early atherosclerosis without endothelial injury group that received a high-fat diet did show a significant increase compared to the control group (3.58 ± 1.71 vs 1.3 ± 0.48, p < 0.001). Similarly, the number of CD31-positive adventitial microvessels in the early atherosclerosis without endothelial injury group that received a high-fat diet was also higher compared to the control group (6.21 ± 3.52 vs 1.90 ± 0.10, p < 0.001).

**Figure 5:**
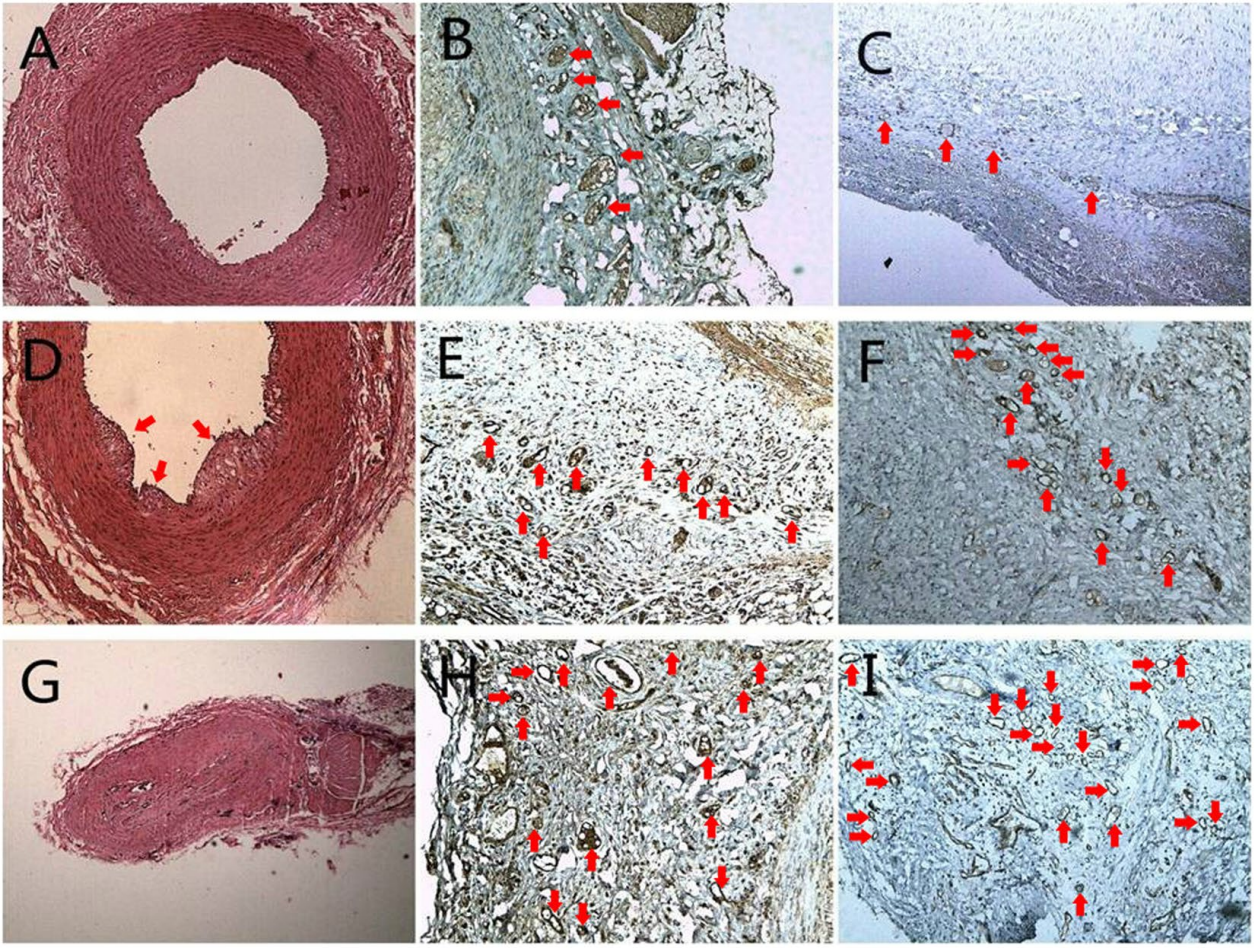
Haematoxylin and eosin stained vessel in early atherosclerosis of the high-fat diet group showed fibre hyperplasia and increased new vessels, ×40 (**A**). Immunohistochemical stain for VEGF of the same vessel showing increased adventitial microvessels, ×100 (**B**). Immunohistochemical stain for CD31 of the same vessel showing many adventitial microvessels, ×100 (**C**). Haematoxylin and eosin stained vessel showing a small plaque in group 4, ×40 (**D**). Red arrowheads indicate immunohistochemical stain for VEGF of the same vessel showing many adventitial microvessels, ×100 (**E**). Immunohistochemical stain for CD31 of the same vessel showing many adventitial microvessels, ×100 (**F**). Haematoxylin and eosin stained vessel showing advanced plaque leading to lumen occlusion in group 5, ×40 (**G**). Immunohistochemical stain for VEGF of the same vessel showing numerous adventitial microvessels, ×100 (**H**). Immunohistochemical stain for CD31 of the same vessel showing numerous adventitial microvessels, ×100 (**I**). The red arrowheads in **B**, **C**, **E**, **F**, **H** and **I** indicate microvessels.

Representative histological cross-sections from the group with early atherosclerosis (Fig. 5A–C), small plaques (Fig. 5D–F) and large plaques (Fig. 5G–I) are shown in Fig. 5. The plaque in Fig. 5D, positive microvessels of VEGF in Fig. 5B,E and H, and the positive microvessels of CD31 in Fig. 5C,F and I are highlighted by small red arrows. It is obvious that the number of VEGF-positive and CD31-positive microvessels increased with the progression of atherosclerosis.

### Relationship between CEUS imaging and histological data

**Figure 6:**
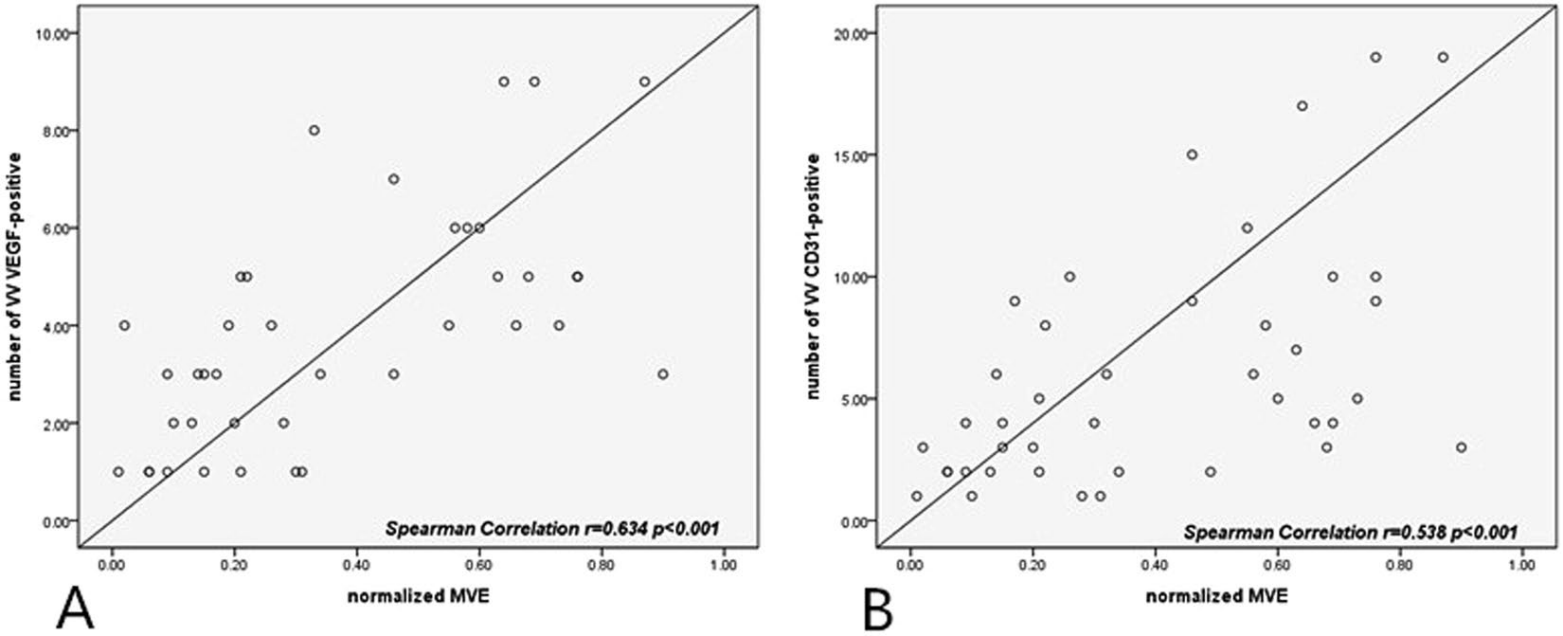
The relationship between the number of VEGF-positive microvessels in the adventitial vasa vasorum and normalized MVE (**A**). The relationship between the number of CD31-positive microvessels in the adventitial vasa vasorum and normalized MVE (**B**).

A positive relationship between the changes in normalized MVE and the number of VEGF-positive microvessels was apparent according to the linear regression analysis (r = 0.634, p < 0.001) (Fig. 6A). Similarly, a linear correlation between the number of CD31-positive microvessels and the normalized MVE was observed (r = 0.538, p < 0.001) (Fig. 6B).

## Discussion

In this study, we explored the feasibility of CEUS imaging for visualizing the neovascularization in the adventitial VV in rabbit models with different degrees of atherosclerosis caused by a high-fat diet with or without balloon injury. The study further confirmed the utilization of quantitative measures of contrast enhancement to measure VV density, especially in early atherosclerosis. Our findings demonstrated that CEUS imaging could be used as a quantitative approach to demonstrate adventitial VV in early atherosclerosis before intimal changes. The contrast-enhanced VV density increased as development of atherosclerosis progressed. This finding was confirmed by histological analysis. The results supported the hypothesis that the adventitial VV network may play an important role in plaque progression. In addition, we designed a model of advanced atherosclerosis with large plaques to observe the adventitial VV and its relationship with the new vessels in plaques. And we found that the enhancement in plaque followed and derived from outer membrane enhancement (Video 2).

Identifying adventitial VV and plaque neovascularization is undoubtedly very important for atherosclerosis progression and plaque vulnerability. Unfortunately, methods for identifying and quantifying VV are limited. Techniques such as coronary angiography and intravascular ultrasound have limitations, including low far-field resolution, low molecular sensitivity, interference by blood, lack of structural definition, and motion and flow artefacts^15^. Nevertheless, CEUS imaging is a non-invasive technique, and because microbubbles could act as the tracer of red blood cells, the microcirculation in the plaque neovascularization and adventitial VV can be clearly visualized. Therefore, CEUS imaging appears to be an emerging technique serving as a valuable method for the early detection of premature atherosclerosis and for the detection of vulnerable plaques in at-risk populations. Some studies have shown that CEUS imaging can be clinically useful in the carotid artery, aorta abdominalis and femoral artery^24–26^. Most of the previously published studies used a qualitative scale to score the presence and amount of intraplaque neovascularization^16, 20, 27^. Quantitative measurements of the contrast enhancement should further improve the reproducibility of the results and reduce observer variability. Recently, a study conducted by Moguillansky *et al*. showed that CEUS imaging can be used for quantifying plaque neovascularization^15^. In the current study, we demonstrated that it can be used for assessing plaque-specific atherosclerosis and the degree of atherosclerosis. We also depicted a relationship between VV neovascularization and atherosclerosis progression *in vivo*.

The VV is a network of small blood vessels with thin walls that are located in the adventitia of large arteries. VV are supposed to carry nutrients to parts of the vascular walls that are distant from the lumen^28^. In the absence of atherosclerosis, the VV is limited to the adventitia and outer media^29^. Arterial regions with low VV density are prone to forming initial plaques, whereas advanced lesions develop more rapidly in regions with high intraplaque VV^30, 31^. VV density is dynamic, increasing with hypercholesterolaemia^32^ and decreasing with cholesterol-lowering strategies like statins^23^. Therefore, the present study focused on adventitial VV, a potential marker of plaque vulnerability. A growth in VV density has been reported to precede intimal thickening and even endothelial dysfunction in animal models^11^ and humans^10^, suggesting that neoangiogenesis could occur at the earliest stage of atherogenesis. However, there has been no study of using CEUS imaging as a marker of early atherosclerosis. In our study, we designed a model of feeding only a high-fat diet to rabbits without injuring the endothelium, and the data suggest that the outer membrane was enhanced in the high-fat diet early atherosclerotic rabbit model, and an increase in adventitial VV without endothelium injury was confirmed by histological staining and immunohistochemistry.

In the present study, we observed adventitial VV in early atherosclerosis without endothelial injury to assess the application and reliability of CEUS imaging to quantify neovessels. In addition, contrast ultrasound was performed to measure adventitial VV and neovessels in plaques of rabbit models with different degrees of atherosclerosis. We found that peak video-intensity is linearly correlated with the histologic indices of VV density. The highlight of our study was that we verified that formation of atherosclerotic plaques was preceded by neoangiogenesis in the adventitia. This conclusion was first drawn by visualizing the increased adventitial VV density in rabbit models generated using high-fat diet without arterial endothelium injury and then further confirmed by histological staining and immunohistochemical examination. Undoubtedly, this information is good news for patients with early-stage carotid atherosclerosis. CEUS imaging may be used as a non-invasive instrument for identifying early atherosclerosis in humans. Further studies in larger cohorts will be needed to confirm the diagnostic value of CEUS imaging in human early-stage carotid atherosclerosis.

Another unique feature of this current study was that we designed a model of atherosclerotic rabbits with lumen occlusion to observe the relationship between the adventitial VV and neovessels in plaques. We found that the enhancement in the outer membrane was earlier than that in plaques and the enhancement from the outer membrane into plaques. This finding may support the view that intraplaque neovascularization comes from the sprouting of the existing VV network in the adventitia^33–35^.

The limitations of this study need to be mentioned. First, no animal model completely mimics human atherosclerosis. Thus, careful extrapolation of our results to humans should be made. Second, our image acquisition relied on 2D long-axis imaging of vessels that does not fully capture the spatially heterogeneity and asymmetric process of atherogenesis, and this issue may lead to imperfect correlations between imaging and histology data.

## Conclusion

The present study demonstrated the feasibility of CEUS imaging for quantifying the VV in early atherosclerosis. The CEUS peak video-intensity predicts the extent of neovascularization, and it was histologically confirmed that the progression of atherosclerotic plaques was related to the VV. Our study also showed that CEUS imaging can be used as a non-invasive quantification tool for the VV. Early atherosclerosis could be identified through this method, and it maybe be helpful for clinical treatment.

## Electronic supplementary material

Video 1

Video 2

SUPPLEMENTARY INFORMATION
