# Supplementary material for: Contrast-Enhanced Ultrasound Imaging Quantification of Adventitial Vasa Vasorum in a Rabbit Model of Varying Degrees of Atherosclerosis
Source: Sci Rep. 2017 Aug 1;7:7032. doi: 10.1038/s41598-017-06127-w (PMC5539241; doi:10.1038/s41598-017-06127-w)
Supplement: Supplementary file 3 — SUPPLEMENTARY INFORMATION [file 41598_2017_6127_MOESM3_ESM.doc]

**Contrast-Enhanced Ultrasound Imaging Quantification of Adventitial Vasa Vasorum in a Rabbit Model of Varying Degree Atherosclerosis**

Xiaoying Li^1#^; Ruyou Zhang^1#^; Zongmin Li^2^; Chunping Ning^3^; Zhenzhen Wang^1^; Meizheng Dang^1^; Yanqing Peng^1^; Xuesong Han^1^; Litao Sun^1🖂^; Jiawei Tian^1🖂^

^1^ The 2nd Affiliated Hospital of Harbin Medical University

^2^ Harbin the first hospital

^3^ The Affiliated Hospital of Qingdao Medical University

^#^ The authors are contribute equally as first authors

^🖂^Corresponding author

**Corresponding author:**

Litao Sun:

Institution: Department of Ultrasound, The 2nd Affiliated Hospital of Harbin Medical University, Harbin 150001, P. R. China

Address: No. 246, Xuefu Road, Nangang Dis, Harbin, Heilongjiang, China

Mail: [litaosun1971@aliyun.com](mailto:litaosun1971@aliyun.com)

Fax: 86-0451-86675845

Tel: 86-0451-86605811

Jiawei Tian:

Institution: Department of Ultrasound, The 2nd Affiliated Hospital of Harbin Medical University, Harbin 150001, P. R. China

Address: No. 246, Xuefu Road, Nangang Dis, Harbin, Heilongjiang, China

**Video legends**

Video 1: This video was taken from a rabbit of group 4, which were modeled by accelerated atherosclerosis. CEUS imaging was in the left side of the screen in the video, and 2-D ultrasound imaging was in the right side of the screen in the video. The carotid artery lumen was dark before contrast injection. After contrast agent filling, more enhanced signals were detected in the adventitia as the arrows in the video. And we can see a small filling defecting on the intima of the posterior wall of the carotid artery.

Video 2: The Video 2 was taken from the accelerated atherosclerotic rabbit group 5. In this video, we can see a hypoechoic plaque almost full of the lumen before the injection of contrast agents in the right side of the screen. After the injection, adventitial contrast was visible in the left side of the screen. And then some enhanced signals were discovered in the plaque from the adventitia.
